# Purging Deleterious Mutations under Self Fertilization: Paradoxical Recovery in Fitness with Increasing Mutation Rate in *Caenorhabditis elegans*

**DOI:** 10.1371/journal.pone.0014473

**Published:** 2010-12-31

**Authors:** Levi T. Morran, Aki H. Ohdera, Patrick C. Phillips

**Affiliations:** Center for Ecology and Evolutionary Biology, University of Oregon, Eugene, Oregon, United States of America; BC Centre for Excellence in HIV/AIDS, Canada

## Abstract

**Background:**

The accumulation of deleterious mutations can drastically reduce population mean fitness. Self-fertilization is thought to be an effective means of purging deleterious mutations. However, widespread linkage disequilibrium generated and maintained by self-fertilization is predicted to reduce the efficacy of purging when mutations are present at multiple loci.

**Methodology/Principal Findings:**

We tested the ability of self-fertilizing populations to purge deleterious mutations at multiple loci by exposing obligately self-fertilizing populations of *Caenorhabditis elegans* to a range of elevated mutation rates and found that mutations accumulated, as evidenced by a reduction in mean fitness, in each population. Therefore, purging in obligate selfing populations is overwhelmed by an increase in mutation rate. Surprisingly, we also found that obligate and predominantly self-fertilizing populations exposed to very high mutation rates exhibited consistently greater fitness than those subject to lesser increases in mutation rate, which contradicts the assumption that increases in mutation rate are negatively correlated with fitness. The high levels of genetic linkage inherent in self-fertilization could drive this fitness increase.

**Conclusions:**

Compensatory mutations can be more frequent under high mutation rates and may alleviate a portion of the fitness lost due to the accumulation of deleterious mutations through epistatic interactions with deleterious mutations. The prolonged maintenance of tightly linked compensatory and deleterious mutations facilitated by self-fertilization may be responsible for the fitness increase as linkage disequilibrium between the compensatory and deleterious mutations preserves their epistatic interaction.

## Introduction

Although mutations are an essential component of adaptive evolution, most mutations that affect fitness are deleterious [1], [2]. As mutations arise in the genome, selection acts to remove deleterious mutations segregating within natural populations. However, if selection is weak or the expression of a mutation is masked by a dominant allele, then deleterious mutations can accumulate in the population over time [3], [4]. Despite their negative effects on fitness, deleterious mutations are capable of drifting to fixation in small populations [5]–[9]. The collective effect of fixing multiple deleterious mutations can drastically reduce the mean fitness of a population, particularly if the mutations interact in a negatively synergistic fashion [8], [10], [11]. Under extreme circumstances, the process of fixing deleterious mutations and subsequent fitness decline can perpetuate itself and eventually drive extinction [7], [8], [12]–[14]. Therefore the ability to curb the accumulation of deleterious mutations is essential for long-term population viability.

Mating systems dictate the way in which mutations are partitioned among offspring and therefore can have a profound influence on mutation accumulation from generation to generation. Organisms that reproduce through self-fertilization are thought to be at a lower risk of accumulating mutations as compared to outcrossing or asexual organisms because selfing promotes the expression of recessive alleles, which in turn makes these alleles more visible to natural selection, allowing them to become “purged” from the population [15]–[17]. The efficacy of this purging will depend on the rate and effect sizes of new mutations. Given the deleterious effects of most mutations, we would predict that increasing the mutation rate should generate progressively larger reductions in fitness, assuming that the mutations accumulate in the genome and that the effects of the mutations are additive or act synergistically [8]. This prediction is generally upheld by most studies that have examined the fitness effects of elevated mutation rates [2], [14], [18]–[20]. However, if selfing facilitates efficient purging, then selfing organisms may be capable of absorbing increased mutation rates with few fitness consequences. Conversely, if the influx of deleterious mutations were to overwhelm the purging process by preventing the production of offspring free of newly arisen deleterious mutations, then selfing organisms would be at risk of fixing increasing numbers of deleterious mutations and become subject to a mutation meltdown [21], [22].

The potential for mutation meltdown is contingent upon the nature of the epistatic interactions between individual deleterious mutations. The potential influence of two interacting loci is strongly affected by the pattern of genetic linkage between the loci. Interacting loci in tight linkage are more likely to be inherited together, increasing their impact on fitness. Because obligate selfing increases the frequency of homozygous loci within a genome, the efficacy of recombination within a selfing lineage becomes limited due to the loss of allelic variants [22], [23]. Therefore selfing lineages generally maintain large portions of their genome in linkage disequilibrium [24]. This aspect of selfing should be beneficial when epistatic interactions between mutations reduce their collective effect on fitness, as is the case with compensatory mutations [10], [25]. However, such tight linkage can potentially limit the effectiveness of purging via selfing if deleterious mutations at multiple loci are too numerous to segregate out among a fixed number of offspring [22]. If mutations have accumulated within most genomes in a population, then genetic hitchhiking, facilitated by linkage, could potentially fix deleterious mutations [6], [7], [21], [26]. Deleterious mutations that escape purging can be carried to fixation simply because of their association with genomes that have high relative fitness in the population.

We used the predominantly selfing nematode *Caenorhabditis elegans* to test the efficacy of purging and the role of linkage in populations exposed to a range of elevated mutation rates. *C. elegans* is an androdioecious soil nematode with hermaphrodites that reproduce through self-fertilization or outcross with males [27]. Importantly, hermaphrodites are incapable of outcrossing with one another. We were able to use a male-lethal mutation (*xol-1*; [28]) to enforce obligate self fertilization on *C. elegans* populations and elevated mutation rates by treating populations with ethyl methanesulfonate (EMS). We maintained all experimental populations under static laboratory conditions, which were comparable to the environment experienced by their ancestral populations, to minimize the potential for laboratory adaptation. We found that while increases in mutation rates easily overcame any presumed benefits of purging, very high mutation rates yielded an unexpected increase in fitness. This unexpected fitness increase may be the direct result of a previously undocumented interaction between genetic linkage maintained by obligate self-fertilization and increasing rates of beneficial or compensatory mutations at very high mutation rates.

## Results

### Mutation rate

**Figure 1:**
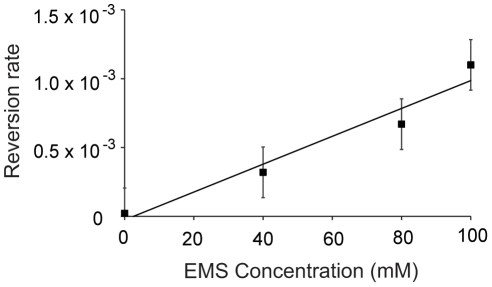
EMS induced mutation rates. Replicate populations of the uncoordinated mutant strain CB665 were mutagenized with a range of EMS concentrations, the F1 generation scored for reversion of the uncoordinated phenotype, and reversion rates calculated for each EMS concentration. The mean induced mutation rate scaled linearly with EMS concentration (R^2^ = 0.26, F_1,196_ = 68.62, P<0.001) Error bars represent two standard errors of the mean.

In *C. elegans*, Rosenbluth and colleagues [18] demonstrated that induced mutation rates increase exponentially with increasing EMS concentrations at low concentrations (0 mM EMS to 30 mM EMS) and then increase linearly at greater concentrations (up to 60 mM EMS). We tested this relationship at higher concentrations, calculating the relative EMS induced mutation rates at 0 mM EMS, 40 mM EMS, 80 mM EMS, and 100 mM EMS by measuring the reversion rate of *C. elegans* mutants exposed to each EMS concentration. Based on reversion rate measurements, we also found that the mutation rate increased linearly with increasing EMS concentration (Figure 1; R^2^ = 0.26, F_1,196_ = 68.62, P<0.001, the R^2^ value is low due to the substantial number of populations that did not exhibit reversion).

### Toxicity

**Figure 2:**
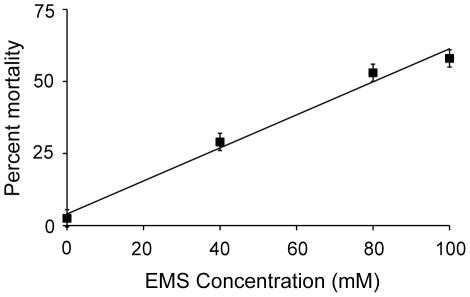
EMS induced mortality rates. Replicate populations of PX385 were exposed to different EMS concentrations. Following mutagenesis the populations were scored for live and dead worms and the mean mortality rate calculated for each EMS concentration. Overall, the EMS induced mean mortality rate, or toxicity, increased linearly (R^2^ = 0.95, F_1,38_ = 682.1, P<0.001) with increasing EMS concentration. Error bars represent two standard errors of the mean.

The direct toxicity of EMS also increases linearly with increasing EMS concentration (Figure 2; R^2^ = 0.95, F_1,38_ = 682.1, P<0.001). Although generally considered among the most benign mutagens, EMS is quite toxic at high concentrations, inducing greater than 50% mortality rates (Figure 2).

### Dose response curves

**Figure 3:**
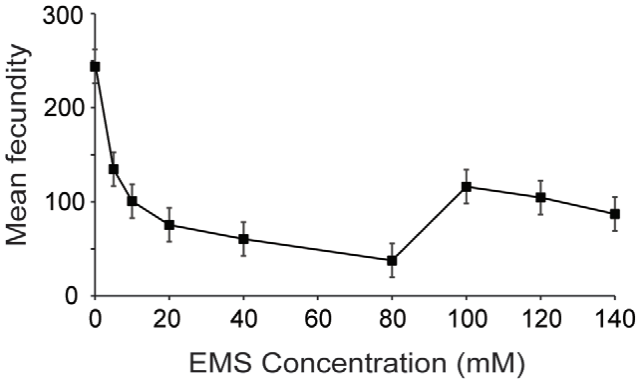
EMS dose response curve. Replicate populations of PX384 were exposed to five generations of mutagenesis across a range of different EMS concentrations. Mean fecundity generally decreased with increasing EMS concentration, however, exposure to 100 mM significantly elevated fecundity relative to much lesser concentrations of EMS (20 mM) (P<0.001, Tukey's HSD). Error bars represent two standard errors of the mean.

Any potential purging of deleterious mutations should be revealed by tracking fitness changes within large, self-fertilizing populations over a number of generations. Populations that successfully purge new mutations should not decline in fitness over time. When exposed to increasing EMS concentrations up to 80 mM for five generations of mutagenesis, obligate selfing *C. elegans* populations exhibit progressively lower fecundities (Figure 3; F_8,486_ = 42.13, P<0.001). However, populations exposed to EMS concentrations greater than 80 mM exhibit surprisingly high fecundity under the same experimental regime (Figure 3). Fecundity after exposure to 100 mM of EMS is comparable to fecundity after exposure to 5 mM EMS and 10 mM EMS (Figure 3; P>0.05, Tukey's HSD) and significantly greater than fecundity after exposure to 20 mM EMS, 40 mM EMS, and 80 mM EMS (Figure 3; P<0.001, Tukey's HSD).

To test the possibility that pleiotropic effects of *xol-1* might be driving our results (Figure 3), we generated another EMS dose response curve using highly selfing wildtype N2 populations, the same genetic background without the *xol-1* mutation. Although males were produced in these populations, we manually removed them before mating, thereby limiting outcrossing rates to substantially less than 1%. We again found that purging was overwhelmed by elevated mutation rates (mean fecundity 0 mM  = 113, mean fecundity 40 mM  = 41, mean fecundity 100 mM  = 102; F_4,274_ = 28.66, P<0.001) and observed an increase in fitness at 100 mM of EMS (P<0.001, Tukey's HSD).

**Figure 4:**
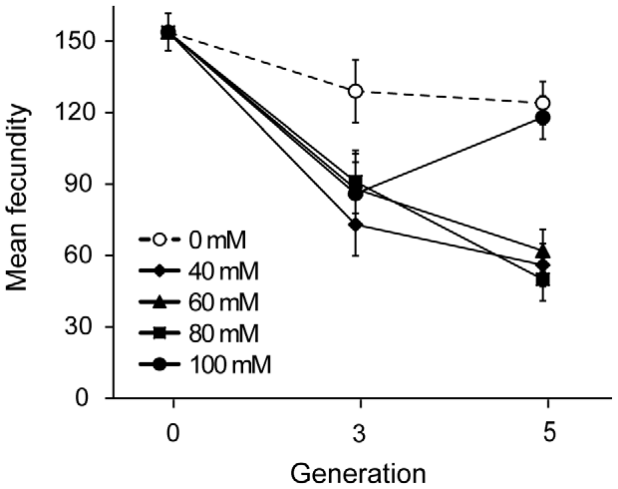
Time series EMS dose response curve. Replicate populations of PX385 were exposed to five generations of mutagenesis across a range of different EMS concentrations. Mean fecundity was assessed prior to mutagenesis, after three generations of mutation, and after five generations of mutation. The mutated populations (solid lines) exhibited reduced mean fecundity, relative to the control populations (dashed line), after three generations (F_1,216_ = 35.64, P<0.001). Then, after five generations, the populations exposed to 100 mM exhibit a substantial increase in mean fecundity while all of the other mutated populations exhibit further reductions in mean fecundity (P<0.001, Tukey's HSD). Error bars represent two standard errors of the mean.

To further test the role of genetic background, we mutagenized a highly divergent natural isolate (CB4856 from Hawaii) carrying the *xol-1* mutation. After three generations of mutation accumulation we found that purging was again overwhelmed at all concentrations (Figure 4 F_4,216_ = 10.01, P<0.001). However, prolonging the experiment to five generations of exposure to EMS, as in our previous dose response curves, generated a fitness increase at 100 mM (Figure 4; P<0.001, Tukey's HSD), while all other concentrations continued to decline in fitness (Figure 4). Additionally, the obligate selfing CB4856 populations maintained at natural mutation rates lost fitness over time (Figure 4; P<0.001, Tukey's HSD). Therefore, (1) failure to purge deleterious mutations and the ability to recapitulate the unexpected fitness increase at high mutation rates were not dependent upon genetic background, and (2) the fitness increase is a cumulative effect of multiple exposures to 100 mM EMS.

**Figure 5:**
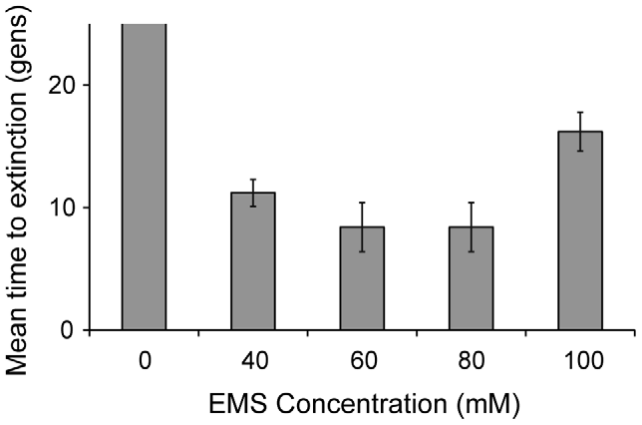
EMS induced extinction rates. Replicate populations of PX385 were continually exposed to a range of different EMS concentrations and driven to extinction. We calculated the mean time to extinction for each EMS concentration. Treatment with 100 mM EMS required more generations of exposure to induce extinction than all other EMS treatments (P<0.001, Tukey's HSD). Control populations, with no EMS exposure, did not go extinct during the course of the experiment. Error bars represent two standard errors of the mean.

These effects persist over prolonged exposure to the mutagen. Populations exposed to 100 mM EMS endured significantly more generations of mutation before going extinct as compared to populations exposed to lesser concentrations of EMS (Figure 5; P<0.001, Tukey's HSD). The survival time of populations exposed to 100 mM EMS was twice that of populations exposed to 80 mM EMS (Figure 5). Thus the fitness increase at 100 mM is not transient, but persists over time.

### Possible selection for EMS resistance

**Figure 6:**
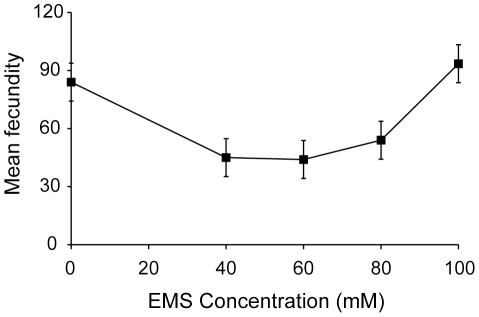
Recapitulated EMS dose response curve. Populations of PX385 that were previously exposed to 100 mM for five generations of mutation, and exhibited a fitness increase, were split into replicate populations and exposed to another five generations of mutation at a range of different EMS concentrations. After the second mutagenesis regime the populations exposed to 100 mM again exhibited increased fitness relative to the other mutagenized populations (P<0.001, Tukey's HSD). Error bars represent two standard errors of the mean.

Might the populations exposed to high concentrations of EMS have simply evolved resistance to EMS itself? We tested this hypothesis by exposing CB4856 populations that had previously exhibited the fitness increase at 100 mM to a range of different EMS concentrations for five generations of mutagenesis. If resistance had evolved, we would expect a decreased influence of EMS at all concentrations. Instead, the dose response curve we generated from “pre-adapted” populations (Figure 6) closely resembled our original dose response curve (Figure 3). The populations exposed to 100 mM EMS exhibited a fitness increase relative to the other mutagenized populations (Figure 6; P<0.001, Tukey's HSD), and had a higher, but not significantly different, mean fitness than populations that were not mutagenized for the second dose response curve (Figure 6; P>0.05, Tukey's HSD). In other words, further exposure to 100 mM EMS generated greater mean fitness than no further exposure to EMS. Therefore, the fitness increase at 100 mM is not the product of evolved EMS resistance in populations exposed to 100 mM EMS.

## Discussion

Mutations and mating systems can interact in multiple ways. Most of the work in mating system theory has focused on single locus effects and the fact that potential inbreeding depression can be “purged” from selfing populations [15]–[17]. Purging can prevent fitness loss only if all newly arising deleterious mutations are removed from a lineage. The efficacy of purging is therefore limited by the total number of genotypes that can segregate within an individual cross, and thus mutations at multiple loci within a lineage have the potential to overwhelm purging and to drive the fixation of deleterious mutations [22]. The interaction between mutation and mating system can also be mediated via the linkage relationship among loci. Linkage can permit deleterious mutations that accumulate within a population to sweep to fixation through genetic hitchhiking [16], [23], [26]. However, the high levels of linkage disequilibrium generated by selfing may also facilitate the fixation of compensatory alleles that modify the effects of the initial mutation [25], [29]. The increased likelihood of linkage disequilibrium among loci means that the epistatic interactions required to maintain the fitness benefits of compensatory mutation are more likely to be maintained in selfing populations relative to outcrossing populations. We find that the balance between these single and multiple locus effects depends strongly on mutation rate.

### Purging in obligate selfing populations

As evidenced by the loss of fitness at all EMS concentrations, we see that purging in obligate selfing *C. elegans* populations is easily overwhelmed by elevated mutation rates (Figure 3). In fact, even marginal increases in mutation rate are capable of overwhelming purging in obligate selfing populations under strong selection against mutation accumulation [14]. Therefore, the efficacy of purging as a mechanism for preventing mutation accumulation may be quite limited, particularly when dealing with mutations of small to moderate effect size at multiple loci.

Morran et al. [14] found that obligate selfing *C. elegans* populations with an N2 background maintain fitness over time when under selection, and it therefore appears that purging at natural mutation rates may be sufficient to prevent mutation accumulation in N2. However, both this study (Figure 4) and our previous work [14] demonstrate that obligate selfing populations with a CB4856 background gradually lose fitness over time, even in the absence of a mutagen. Differences between these strains may be the result of a greater natural mutation rate and/or reduced mutational robustness in the CB4856 strain relative to N2. Such differences in mutational decay have previously been identified in *C. elegans* and among several other nematode species [30]. Interestingly, CB4856 naturally maintains much greater outcrossing rates than the N2 strain [31] and does not lose fitness under conditions that permit outcrossing [14]. Unlike selfing, outcrossing is capable of breaking apart groups of linked genes, thus reducing the probability of fixing accumulated mutations [32], [33] or reducing an existing genetic load [34]. It may be that once purging is overwhelmed by mutations at multiple loci, the linkage disequilibrium generated by selfing traps populations at a level of reduced fitness.

### Fitness increase at high mutation rate

Given that purging was overwhelmed, it is perhaps not surprising that we found that subsequent increases in mutation rate (Figure 1) generally led to significantly larger reductions in fitness (Figure 3). However, contrary to expectation, we identified a non-monotonic fitness response generated by a remarkably high mutation rate (Figure 3). By measuring fitness in four separate dose response curve experiments (Figure 3, 4, 6) and measuring extinction rates in populations with prolonged EMS exposure (Figure 5), we find that populations in each experiment exhibit a relative increase in fitness after regular exposure to 100 mM EMS.

The increase in fitness is a cumulative result of several exposures to 100 mM EMS, as three generations of mutagenesis were insufficient to drive the fitness increase (an effect that was replicated under a variety of experimental conditions; Figure 4). Therefore the increase must be driven by a mechanism with cumulative effects, such as mutation accumulation. We tested several aspects of our experimental design to identify a mechanism driving the increase. We see that 100 mM EMS induces greater mutation rates (Figure 1) and equal or greater toxicity than lesser concentrations of EMS (Figure 2). Therefore, the fitness increase is not a direct product of altered mutagenic properties of EMS at 100 mM. The fitness increase occurs in N2 populations both with (Figure 3) and without the *xol-1* mutation, ruling out the possibility of pleiotropic effects of the *xol-1* mutation. Further, the fitness increase is present in populations with either an N2 (Figure 3) or CB4856 (Figure 4) genetic background. Finally we found that the fitness increase is not the product of selection during the course of the experiment, as exposure to 100 mM did not make the populations more resistant to EMS (Figure 6).

Although recombination rate may scale with EMS concentration, as EMS exposure is known to increase recombination rates in some species [35], [36], it is unlikely that purging facilitated by elevated recombination rates explains the fitness increase at 100 mM. If purging were more effective at 100 mM, we would expect a slow decline in the fitness of populations treated with 100 mM as fewer mutations would accumulate each generation relative to all other treatments. However, we see a rapid decline in fitness and then a sharp rebound (Figure 4), with the fitness increase being the cumulative result of multiple exposures to EMS. Further, greater recombination rates may not increase the efficacy of purging at such high mutation rates. Recombination can only shuffle alleles, and when overwhelmed by mutations, additional shuffling of alleles may only serve to shuffle mutant alleles with other mutant alleles. It is more likely that segregation, rather than recombination rate, is the factor limiting purging at such high mutation rates. Therefore, it seems probable that the fitness increase generated by exposure to 100 mM EMS is the product of unexpected genomic consequences of high mutation rates coupled with the genomic effects of self-fertilization.

The prediction that large increases in mutation rate cause major reductions in fitness is based on the supposition that most mutations with fitness effects are deleterious and that the effects of these deleterious mutations are either additive or negatively synergistic. However, compensatory mutations increase fitness by interacting epistatically with deleterious mutations in the genome [10], [25]. Therefore, a compensatory mutation itself may have little or perhaps negative fitness effects, but that same mutation has positive fitness effects when expressed in a specific genetic background. Silander et al. [37] demonstrated that deleterious mutations can be context-dependent: as fitness declines, the ratio of beneficial to deleterious mutations increases (see also [38]–[40]). Compensatory mutation has also been shown to facilitate substantial fitness recovery in mutation accumulation lines and natural populations previously overwhelmed by accumulated deleterious mutations [41]–[43]. Therefore, the classification of mutations as deleterious depends greatly upon the genetic background into which that mutation is incorporated, and fewer mutations have deleterious effects in genetic backgrounds with poor fitness [44]. So, although elevating the mutation rate may reduce fitness through the influx of deleterious mutations, as fitness declines the ratio of beneficial to deleterious mutations may shift to a point at which a significant proportion of new mutations are beneficial, or compensatory, and their collective effects begin to elevate fitness. Such an effect would be most pronounced at high mutation rates and would likely only materialize after several generations of mutation accumulation.

Self-fertilization plays a critical role in this scenario, because selfing greatly increases the likelihood that the two mutations will be found in the same genetic background. All of the experimental populations utilized in this study reproduced either predominantly or solely through self-fertilization. The widespread homozygosity resulting from prolonged periods of selfing is a very effective means of maintaining linkage groups [24], especially those favored by selection. If exposure to 100 mM EMS were to increase the rate of compensatory mutation relative to lesser EMS concentrations, then selfing would likely permit the epistatic interactions between loci to be maintained for many generations [22], [23], [26], [32], [33].

Regardless of the mechanism driving the fitness increase exhibited by populations exposed to 100 mM EMS, the result is a testament to the resiliency of the genome. Consistent exposure to high mutation rates should wreak havoc on the genome, and repeated exposure to 80 mM EMS (Figure 5) appears to do just that. However, the genome is able to recover a large proportion of the fitness lost at 80 mM EMS when exposed to 100 mM EMS (Figure 3). This result is quite surprising and challenges the long-held beliefs concerning the relationship between mutation rates and fitness.

## Materials and Methods

*C. elegans* strains N2 and CB4856 are stock populations originally derived from single individuals that were isolated from natural populations. The N2 strain, from Bristol, England, has been maintained in the laboratory for approximately forty years and naturally exhibits very low male frequencies and therefore maintains low outcrossing rates (<1%) [31]. CB4856, however, is a more recent isolate from Hawaii, USA, and maintains significantly greater male frequencies and outcrossing rates (∼10–25%) than N2 [31]. The N2, CB4856, and CB665 strains were obtained from the Caenorhabditis Genetics Center (University of Minnesota, Minneapolis, MN). Development of the *xol-1(y9)* mutation containing PX384 and PX385 strains is described in [14] (Table 1). *xol-1* expression activates X-chromosome dosage compensation in *C. elegans*, which occurs normally in hermaphrodites as they possess two copies of the X-chromosome [28]. However, males possess only a single copy of the X-chromosome, therefore dosage compensation resulting from *xol-1* expression is lethal for them, thereby enforcing obligate self fertilization within these populations.

**Table 1:** Dose response curve experimental design.

Dose response curve	*C. elegans* strain	Genetic background	*xol-1*	EMS concentration (mM)	Replicate populations per Concentration
1	PX384	N2	Yes	0, 5, 10, 20, 40, 80, 100, 120, 140	4
2	N2	N2	No	0, 40, 100	3
3	PX385	CB4856	Yes	0, 40, 60, 80, 100	5
4	PX385Previously mutated at 100 mM	CB4856	Yes	0, 40, 60, 80, 100	5

All populations were reared on agar plates constructed by pouring 24 mL of autoclaved NGM Lite (US Biological, Swampscott, MA) into a 10 cm Petri dish. Each plate was seeded with 5 µL of OP50 *Escherichia coli*, and all populations were maintained at 20°C.

### Dose response curves

All dose response curve experiments (Table 1) were conducted by exposing approximately one thousand individuals (or the entire population if the census size dropped below one thousand individuals) from each replicate population (Table 1) to a specific concentration of ethyl methanesulfonate (EMS, cat. #M0880, Sigma-Aldrich, St. Louis, MO) every other generation for ten generations or five total mutagenesis events [45]. EMS is commonly used to elevate mutation rates in a wide variety of organisms due to its limited toxicity (relative to other mutagens) and its tendency to induce point mutations, particularly A/T to G/C transitions [34], [45], [46]. Another valuable aspect of EMS mutagenesis is that induced mutation rates are positively correlated with increasing EMS concentration, therefore mutation rates can be titrated through differing amounts of EMS exposure [18]. Control or “0” mM populations were subject to the same buffer and mixing procedures as mutated populations, with no EMS added during mixing. Populations were chunk transferred to freshly seeded plates in the off, non-mutagenic generations [47].

Following three to five generations of mutagenesis (depending on the treatment; Table 1) and a recovery period of at least two generations, the mean lifetime self-fecundity of fifteen to twenty arbitrarily sampled L4 (late stage larval) individuals was measured for each experimental treatment. Mean lifetime fecundity serves as a proxy for overall fitness in *C. elegans* [30]. Single worms were picked to 35×10 mm plates seeded with OP50 and allowed to self. We then counted the total number of offspring per worm four days after picking, allowing time for the offspring to mature to the L3 larval stage, therefore incorporating offspring survival from egg to L3 into our fecundity counts. Individuals that did not produce offspring were counted as having a mean fecundity of zero if the presence of worm tracks indicated that the worm was not killed during transfer. Otherwise worms with no offspring or tracks were excluded from our analysis. The effects of EMS concentration on mean fecundity were tested using an ANOVA (JMP-IN 5.1, SAS Institute, Cary, NC). Additionally, Tukey's HSD tests were performed post-hoc for specific comparisons between EMS concentrations.

### Extinction rate

Four replicate PX385 replicate populations were exposed to 0 mM, 40 mM, 60 mM, 80 mM, and 100 mM EMS using the same experimental regime employed for the dose response curves. However, instead of ending the experimental regime after five generations of mutagenesis, we extended the duration of the experiment indefinitely and measured the time to extinction for each population. If one thousand worms were not available, we transferred the maximum number possible. The presence of fewer than five worms on a plate was counted as extinction because such low numbers can not be maintained through the buffer and mixing procedures required for EMS mutagenesis [45].

### Toxicity

Ten replicate populations of PX385 with approximately one thousand L4 stage individuals apiece were given a single exposure to 0 mM, 40 mM, 80 mM, or 100m M EMS. After mutagenesis the worms were transferred to a freshly seeded plate and allowed to mature. Mortality was measured by counting a total number of 200 individuals across a transect representing approximately 20% of the plate and scoring individuals as either living or dead (by prodding the worms with a platinum pick and assessing movement). Mortality rates were calculated by determining the frequency of dead worms relative to the total counted. We evaluated the relationship between mean mortality rate and EMS concentration with linear regression analysis and ANOVA (JMP-IN 5.1, SAS Institute, Cary, NC). Both methods yielded the same results; the linear regression data is presented.

### Mutation rate

The mean relative mutation rates of 0 mM, 40 mM, 80 mM, and 100 mM EMS were measured with a mutator assay using the CB665 strain, which possess the *unc-58 (e665)* allele, and therefore exhibit a dominant uncoordinated phenotype that greatly impairs movement [48]. Reversion of the uncoordinated phenotype is caused by intragenic and extragenic suppressor mutations that restore normal movement [48]. Fifty replicate populations of approximately two thousand L4 individuals apiece were mutated for one generation at each designated EMS concentration. Mutagenesis was conducted as described in [45]. The populations were transferred to freshly seeded plates after mutagenesis and allowed to self-fertilize. Their adult offspring were then scored for the presence or absence of individuals with restored movement, thus indicating reversion. Then we calculated the mean mutation rate for each EMS concentration.

Following [45], the total number of mutagenized worms that produced offspring in each population was calculated as:
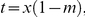
where *t* is the number of mutagenized worms that produced offspring, *m* is the mortality rate specific to each EMS concentration, and *x* is the number of mutagenized individuals.

The estimated number of revertants in each population was calculated as:
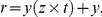
where *r* is the estimated number of revertants and *y* is the measured value of revertants in each population (measured binomially with a value of one indicating the presence of revertants and a value of zero indicating no revertants). The parameter *z* represents the probability of multiple reversions occurring in the same population as calculated for each EMS concentration with fifty populations per concentration:
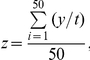



The mutation rate for each population was calculated as:
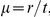
where *µ* is the mutation rate for each population. We then calculated the mean mutation rate for each EMS concentration. We evaluated the relationship between mean mutation rate and EMS concentration with linear regression analysis and ANOVA.
